# Hormonal Correlates and Predictors of Nutritional Recovery in Malnourished African Children

**DOI:** 10.1093/tropej/fmx075

**Published:** 2017-10-30

**Authors:** Helen M Nabwera, Robin M Bernstein, Schadrac C Agbla, Sophie E Moore, Momodou K Darboe, Mariama Colley, Amadou T Jallow, Richard Bradbury, Jennifer Karafin, Anthony J Fulford, Andrew M Prentice

**Affiliations:** 1 MRC Unit The Gambia, Banjul, The Gambia; 2 MRC International Nutrition Group, London School of Hygiene and Tropical Medicine, London, UK; 3 Department of Anthropology, University of Colorado at Boulder, Boulder CO, USA; 4 Division of Women’s Health, King’s College London, London, UK

**Keywords:** hormones, malnutrition, nutritional rehabilitation, Gambian children

## Abstract

**Background:**

Malnourished children show variable growth responses to nutritional rehabilitation. We aimed to investigate whether these differences could be explained by variations in growth and energy-regulating hormones.

**Methods:**

Quasi-experimental study: Children aged 6–24 months in rural Gambia were recruited to controls if weight-for-height *z*-score (WHZ) > −2 (*n* = 22), moderate acute malnutrition if WHZ < −2 and > −3 (*n* = 18) or severe acute malnutrition if WHZ < −3 (*n* = 20). Plasma hormone and salivary CRP levels were determined by ELISA.

**Results:**

In univariable analyses, increases in weight-for-age *z*-score (WAZ) in malnourished children were positively correlated with insulin (*F*-ratio 7.8, *p* = 0.006), C-peptide (*F*-ratio 12.2, *p* < 0.001) and cortisol (*F*-ratio 5.0, *p* = 0.03). In multivariable analysis, only baseline C-peptide (*F*-ratio 7.6, *p* = 0.009) predicted the changes in WAZ over 28 days of interventions.

**Conclusion:**

In rural Gambian, malnourished children, although it cannot be used in isolation, baseline C-peptide was a predictor of future response to rehabilitation.

## BACKGROUND

Growth faltering is endemic in children aged <2 years in sub-Saharan Africa and is associated with high rates of morbidity and mortality [1]. A proportion of these children develop life-threatening severe acute malnutrition (SAM) that requires urgent and intensive health service investment. The causes of the wide variability in the response of children suffering from SAM to nutritional rehabilitation are unknown [2], (except for HIV infection) even when rigorously implemented according to latest international guidelines [2, 3]. The identification of simple prognostic indicators, measurable at initial diagnosis, would greatly assist in triaging malnourished children between those who require high-cost, labour-intensive tertiary-level care and those suitable for community management of acute malnutrition.

Healthy growth is regulated by hormonal pathways that are sensitive to nutritional and infectious stressors [4]. Periods of inadequate energy and nutrient intake or increased metabolism because of infections alter the hormonal regulation of growth by the growth hormone–insulin-like growth factor axis that can shift the timing and duration of the various phases of child growth [5, 6]. Previous studies have evaluated the energy-regulating hormonal changes in children with SAM undergoing intensive inpatient nutritional rehabilitation [7–13].

The primary aim of this study was to investigate whether the high degree of variability in children’s responses to nutritional rehabilitation could be explained and predicted by differences in energy and growth-regulating hormones, to support the development of point-of-care assessment tools that would enable clinicians to optimize the timing of nutrition interventions in malnourished children.

## METHODOLOGY

### Study population

The study was conducted at the Medical Research Council Unit, The Gambia’s rural field station in Keneba. The study participants were children aged 6–24 months who presented to the outpatient clinic. They were assigned to one of the three nutritional groups, according to the World Health Organization (WHO) classifications using weight-for-height *z*-scores (WHZ), mid-upper arm circumference (MUAC) and clinical assessment [14, 15]. Unmatched controls had a WHZ  > −2. Children were excluded if they had significant medical complications requiring resuscitation, were HIV infected or had congenital or chronic medical conditions.

Sixty children were recruited into the study and completed follow-up from June 2013 to October 2014. None of the children with SAM had pedal oedema at presentation and no children died during the study.

### Study design and interventions

A quasi-experimental study design: At baseline, all the children recruited received 20 ml/kg of Formula 75 (Nutriset) as a test meal. Children with moderate acute malnutrition (MAM) and SAM had further test meals on Days 14 and 28. All malnourished children were managed according to WHO and national malnutrition guidelines [15, 16].

Anthropometric measurements including weight, height, head circumference, MUAC and knee–heel length were taken by trained field workers on alternate days for the first 28 days then at 6 months.

### Biological sampling and analysis

At enrolment, pre- and post-test meal venous blood, saliva and urine samples were collected from all the recruited children. Subsequent samples were collected from children with MAM and SAM on Days 14 and 28. The hormone and salivary C-reactive protein (CRP) analyses were performed using ELISA on plasma and saliva, respectively (R&D Systems, Minneapolis, USA; ALPCO, New Hampshire, USA; Merck Millipore, Darmstadt, Germany; Salimetrics, Pennsylvania, USA).

### Statistical analysis

The study sample size of 60 was derived from the reported variability in energy-regulating hormones in malnourished children <5 years over time and between malnourished children and controls, from previous work in this setting (Nweneka, Prentice *et al.*, unpublished) and Stein *et al.*’s data [11]. Where the data were skewed, the Kruskal–Wallis test was used to compare baseline continuous variables (i.e. age, anthropometry, salivary CRP) between the three nutritional groups. The Fisher’s exact test was used to assess the association between the nutritional groups and the categorical baseline characteristics (i.e. gender; diagnoses of diarrhoea or urinary tract infections; antibiotic prescription; parental education).

Wilcoxon signed-rank test was used to compare pre- and post-prandial hormonal levels within each nutritional group at baseline. A mixed-effects model was used to assess differences in hormone levels between nutritional groups at baseline and over time. A piecewise linear random slope model was used to assess the change in anthropometry over time using three time intervals: 0–14 days, 14–28 days and 29–180 days. The Wald test was used to test for interaction between time and nutritional group. Repeated measures ANOVA was used to assess which biochemical indices were good predictors of nutritional recovery. Analyses were conducted using Stata 12.1 (Stata Corp) and DataDesk 7.0.2 (Data Description Inc, Ithaca, NY) (for further details, see Supplementary Material).

## ETHICAL CONSIDERATIONS

The study was approved by The Gambia Government/MRC Unit, The Gambia Joint ethics committee, SCC 1306 and the London School of Hygiene and Tropical Medicine ethics committee. All guardians had the study explained to them in detail by the field staff in their local language and signed a consent form in English.

## RESULTS

### Study population

Diarrhoea was a more common presenting symptom in children with SAM than in the other groups [58 vs. 29% (MAM) and 11% (controls)]; *p* = 0.01 (Table 1).

**Table:** Table 1. Baseline characteristics

	Nutritional category			*p*
	Controls (*N* = 22)	MAM (*N* = 18)	SAM (*N* = 20)	
Age in months, median (IQR)	12.75 (10.2, 19.3)	16.5 (12.0, 22.0)	12.0 (10.3, 16.5)	0.22^a^
Age of weaning in months, median (IQR)	6.0 (5.0, 6.0)	6.0 (6.0, 6.0)	6.0 (5.5, 6.0)	0.82^a^
WHZ, median (IQR)	−1.2 (−1.8, 0.1)	−2.6 (−2.8, −2.1)	−3.4 (−3.9, −3.2)	<0.001^a^
WAZ, median (IQR)	−1.5 (−1.7, −0.1)	−2.8 (−3.1, −2.1)	−3.2 (−3.4, −2.9)	<0.001^a^
HAZ, median (IQR)	−0.7 (−1.8, 0.03)	−1.7 (−2.5, −1.0)	−1.9 (−2.3, −0.9)	0.08^a^
Salivary CRP, ng/ml, median (IQR)	2.9 (2.4, 4.1)	4.9 (2.8, 10.3)	5.6 (4.1, 9.9)	0.04^a^
Urinary tract infections, *n* (%)	4 (19)	2 (12)	1 (6)	0.47^b^
Diarrhoea, *n* (%)	2 (11)	4 (29)	11 (58)	0.01^b^
Antibiotics prescribed	9 (41)	11(61)	18 (90)	0.003^b^
Females, *n* (%)	11 (50)	8 (44)	10 (50)	0.90^b^
Mother had no formal education, *n* (%)	20 (91)	12 (67)	16 (80)	0.17^b^
Father had no formal education, *n* (%)	9 (41)	10 (56)	15 (75)	0.09^b^

IQR, interquartile range; HAZ, height-for-age *z*-score.

a Kruskal–Wallis test.

b Fisher’s exact test.

### Anthropometric changes over time

The change in anthropometric measurements was variable across all nutritional groups. The children with MAM and SAM showed significant catch-up growth between 0 and 14 days in all the anthropometric parameters (Table 2).

**Table:** Table 2. Change in anthropometric measurements by nutritional group^a^

Anthropometry	Controls		MAM		SAM		*p*^b^
	Change per day (95% CI)^a^	*p*	Change per day (95% CI)^a^	*p*	Change per day (95% CI)^a^	*p*	
Weight (kg)							
Within 0–14 days	0.003 (−0.003, 0.01)	0.37	0.03 (0.02, 0.04)	<0.001	0.04 (0.03, 0.04)	<0.001	<0.001
Within 15–28 days	−0.002 (−0.01, 0.005)	0.62	0.002 (−0.005, 0.01)	0.50	0.008 (0.002, 0.01)	0.008	0.08
Within 29–180 days	0.008 (0.006, 0.01)	<0.001	0.005 (0.003, 0.007)	<0.001	0.006 (0.004, 0.007)	<0.001	0.06
MUAC (cm)							
Within 0–14 days	0.008 (−0.003, 0.02)	0.15	0.04 (0.03, 0.05)	<0.001	0.05 (0.04, 0.06)	<0.001	<0.001
Within 15–28 days	−0.002 (−0.01, 0.01)	0.70	0.02 (0.004, 0.03)	0.01	0.01 (0.002, 0.03)	0.02	0.04
Within 29–180 days	0.003 (0.001, 0.004)	<0.001	0.003 (0.001, 0.004)	<0.001	0.003 (0.001, 0.004)	<0.001	0.37
Kneel–heel (cm)							
Within 0–14 days	0.004 (−0.01, 0.01)	0.44	0.02 (0.01, 0.03)	<0.001	0.02 (0.008, 0.03)	<0.001	0.02
Within 15–28 days	0.003 (−0.003, 0.01)	0.34	0.003 (−0.003, 0.01)	0.34	0.003 (−0.003, 0.01)	0.34	0.94
Within 29–180 days	0.01 (0.007, 0.01)	<0.001	0.005 (0.002, 0.008)	0.003	0.009 (0.006, 0.01)	<0.001	0.02
WHZ scores							
Within 0–14 days	−0.02 (−0.03, −0.004)	0.009	0.05 (0.03, 0.06)	<0.001	0.05 (0.04, 0.06)	<0.001	<0.001
Within 15–28 days	−0.004 (−0.02, 0.01)	0.54	−0.004 (−0.02, 0.01)	0.54	−0.004 (−0.02, 0.01)	0.54	0.70
Within 29–180 days	0.004 (0.001, 0.008)	0.03	0.004 (0.001, 0.008)	0.03	0.004 (0.001, 0.008)	0.03	0.24
WAZ scores							
Within 0–14 days	−0.007 (−0.014, <0.001)	0.06	0.036 (0.03, 0.04)	<0.001	0.040 (0.033, 0.047)	<0.001	0.39
Within 15–28 days	−0.008 (−0.015, −0.001)	0.03	−0.007 (−0.016, 0.002)	0.11	0.003 (−0.005, 0.010)	0.52	0.16
Within 29–180 days	<0.001 (−0.0001, 0.002)	0.09	<0.001 ( < −0.001, 0.002)	0.09	<0.001 ( < −0.001, 0.002)	0.09	0.004
HAZ scores							
Within 0–14 days	0.009 (−0.002, 0.020)	0.10	−0.004 (−0.016, 0.009)	0.54	0.015 (0.004, 0.026)	0.01	0.04
Within 15–28 days	−0.009 (−0.019, 0.002)	0.11	−0.006 (−0.019, 0.006)	0.31	−0.005(−0.016, 0.006)	0.36	0.31
Within 29–180 days	−0.001 (−0.002, < −0.001)	0.03	−0.001 (−0.002, < −0.001)	0.03	−0.001 (−0.002, < −0.001)	0.03	0.34

HAZ, height-for-age *z*-score.

a All estimates adjusted for age and gender.

b Interaction test between time and nutritional group.

**Fig. 1.**
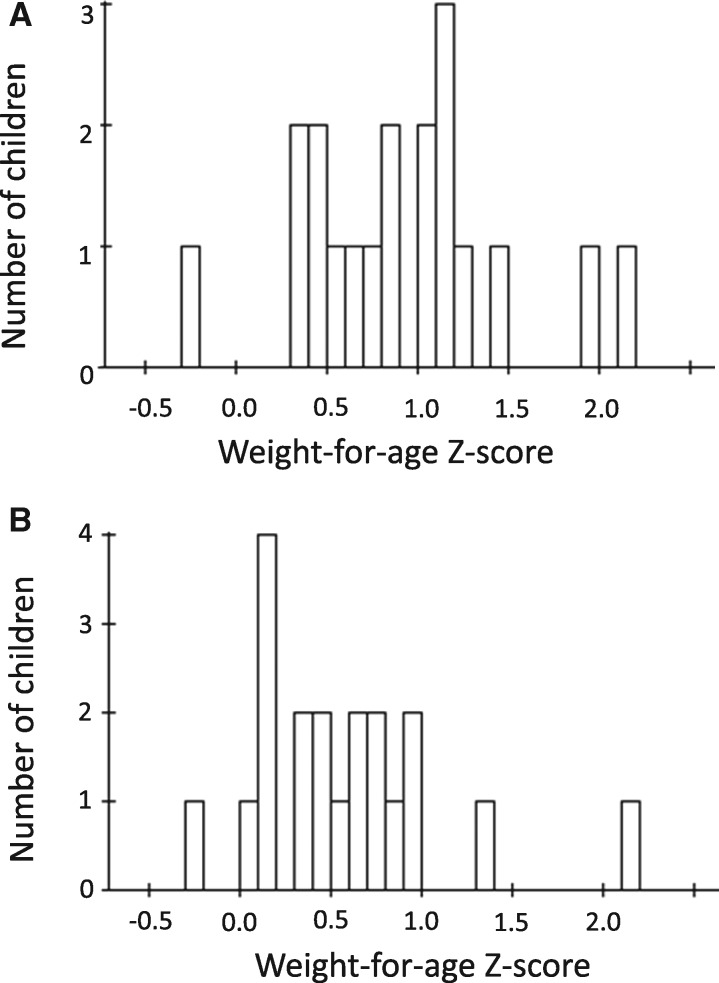
Weight-for-age *Z*-score (WAZ) gain. (**A**) MAM group. Number of children by WAZ gain. (**B**) SAM group. Number of children by WAZ gain.

A key feature of the nutritional recovery in both the MAM and SAM groups was the high degree of variability (Fig. 1). This variability in WAZ recovery was not predicted by baseline age, anthropometry, breastfeeding status, salivary CRP, amount consumed at the test meal, presence of diarrhoea or a urinary tract infection.

### Hormone status at baseline

**Fig. 2.**
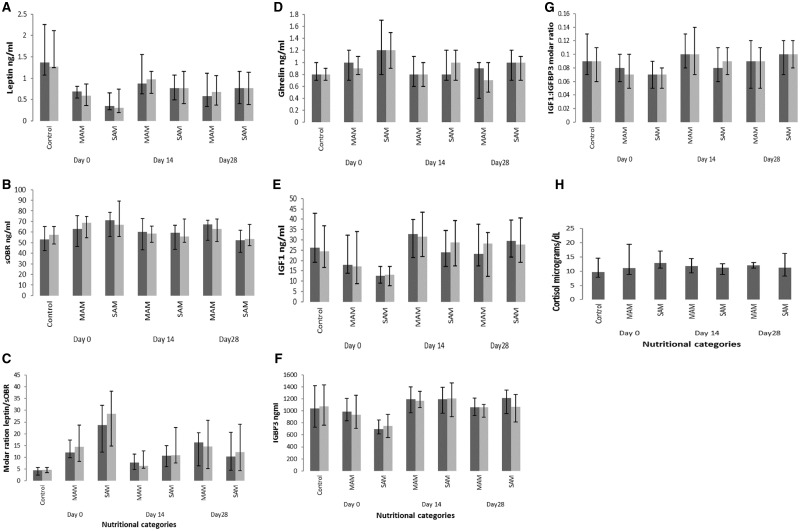
Changes in hormone and receptor levels over time by nutritional category. (**A**) Leptin levels in nanograms per millilitre. Dark grey bars = pre-test meal levels, pale grey bars = post-test meal levels. (**B**) sOBR levels in nanograms per millilitre. Dark grey bars = pre-test meal levels, pale grey bars = post-test meal levels. (**C**) Molar excess of sOBR/leptin. Dark grey bars = pre-test meal levels, pale grey bars = post-test meal levels. (**D**) Total ghrelin in nanograms per millilitre. Dark grey bars = pre-test meal levels, pale grey bars = post-test meal levels. (**E**) IGF-1 in nanograms per millilitre. Dark grey bars = pre-test meal levels, pale grey bars = post-test meal levels. (**F**) IGFBP3 in nanograms per millilitre. Dark grey bars = pre-test meal levels, pale grey bars = post-test meal levels. (**G**) Molar excess of IGF-1/IGFBP3. Dark grey bars = pre-test meal levels, pale grey bars = post-test meal levels. (**H**) Pre-prandial cortisol in micrograms per decilitre.

There was no difference between the pre- and post-prandial levels of any of the hormones in any of the nutritional groups (Fig. 2). The pre- and post-prandial levels were therefore averaged for subsequent analysis.

Compared with controls, the geometric mean ratio of leptin was significantly lower in both MAM {0.4 [95% confidence interval (CI): 0.3, 0.6], *p* < 0.05} and SAM [0.3 (95% CI: 0.2, 0.5), *p* < 0.05]. Insulin-like growth factor 1 (IGF-1) and IGF-binding protein 3 (IGFBP3) were significantly lower in only SAM [0.5 (95% CI: 0.3, 0.7), *p* < 0.05] and 0.7 (95% CI: 0.5, 0.9), *p* < 0.05], respectively. Compared with controls, the molar excess of soluble leptin receptor (sOBR) was significantly higher in both MAM [3.4 (95% CI: 1.9, 6.1), *p* < 0.05] and SAM [ratio 4.9 (95% CI: 2.8, 8.6), *p* < 0.05]. There was no evidence of a difference in cortisol, insulin or C-peptide levels between the groups (Table 3).

**Table:** Table 3. Baseline hormone and receptor levels by nutritional group

Hormone	Control (a)			MAM (b)			SAM (c)			Geometric mean ratio (95% CI) (b vs. a)	Geometric mean ratio (95% CI) (c vs. a)
	Fasted	Fed	*p*^a^	Fasted	Fed	*p*^a^	Fasted	Fed	*p*^a^		
Leptin, ng/ml, median (IQR)	1.4	1.3	0.52	0.7	0.6	0.49	0.4	0.3	0.13	0.4	0.3
	(1.1–2.3)	(1.3–2.1)		(0.5–0.8)	(0.4–0.9)		(0.3, 0.7)	(0.2, 0.7)		(0.2, 0.6)**	(0.2, 0.5)**
sOBR, ng/ml, median (IQR)	53.1	57.5	0.88	62.9	68.6	0.21	71.2	67	0.70	1.2	1.2
	(42.5–65.2)	(48.8–65.7)		(75.3–46.4)	(54.8–74.8)		(55.9–78.6)	(55.8–89.1)		(0.9, 1.4)	(1.0, 1.5)**
Molar excess sOBR/leptin	4.4	4.7	0.43	12.1	14.5	0.06	23.7	28.6	0.07	3.4	4.9
	(2.4–5.6)	(3.5–5.6)		(9.8–17.3)	(8.2–23.7)		(12.2–32.2)	(14.7–38.2)		(1.9, 6.1)**	(2.8, 8.6) **
Total ghrelin ng/ml, median (IQR)	0.8	0.8	0.73	1.0	0.9	0.97	1.20	1.2	0.33	1.3	1.6
	(0.7–1.0)	(0.7–0.9)		(0.7–1.2)	(0.8–1.1)		(0.81–1.70)	(0.9–1.5)		(0.9, 1.8)	(1.1, 2.3)**
IGF-1, ng/ml, median (IQR)	26.2	24.5	0.49	17.8	17.2	0.15	12.5	13.0	0.79	0.7	0.5
	(19.2–42.9)	(16.5–36.9)		(13.8–32.3)	(8.7–34.0)		(9.1–17.0)	(7.8–17.0)		(0.5, 1.1)	(0.3, 0.7)**
IGFBP3, ng/ml, median (IQR)	1039.1	1073.4	0.33	990.5	937.1	0.68	697.2	747.6	0.19	0.9	0.7
	(730.1–1420.7)	(762.7–1430.6)		(834.5–1207.6)	(709.2–1258.2)		(614.0–846.4)	(559.8–944.2)		(0.6, 1.1)	(0.5, 0.9)**
Molar ratio IGF1:IGFBP3	0.09	0.09	0.36	0.08	0.07	0.74	0.07	0.07	0.35	0.8	0.7
	(0.07–0.1)	(0.06–0.1)		(0.06–0.1)	(0.05–0.1)		(0.05–0.09)	(0.05–0.08)		(0.6, 1.1)	(0.6, 0.9)**
Cortisol, µ/dl, median (IQR)	9.7	–	–	11.1	–	–	12.9	–	–	1.2	1.4
	(7.9–14.6)			(8.8–19.5)			(11.1–17.1)			(0.8, 1.7)	(0.9, 1.9)*
C-peptide, pM, median (IQR)	240.7	–	–	212.3	–	–	218.9	–	–	1.1	1.2
	(129.3–309.6)			(154.8–294.7)			(145.7–464.5)			(0.7, 1.8)	(0.7, 2.0)
Insulin, µIU/ml, median (IQR)	2.1	–	–	2.2	–	–	1.1	–	–	1.0	0.7
	(1.1–4.4)			(0–5.9)			(0–3.05)			(0.4, 2.3)	(0.3, 1.7)

IQR, interquartile range;

a Wilcoxon signed-rank test comparing fasted and fed hormonal levels.

* Bonferroni-adjusted *p* < 0.10.

** Bonferroni-adjusted *p* < 0.05.

### Hormone changes over time

The lack of difference in hormone levels between the pre- and post-prandial states persisted at the subsequent time points in the MAM and SAM groups (Fig. 2). In the multivariable analysis, from Days 0 to 14, significant increases in the geometric mean ratios in both MAM and SAM were found for the following: total leptin [1.6 (95% CI: 1.1, 2.4), *p* = 0.002 and 1.8 (95% CI: 1.3, 2.6), *p* < 0.001]; IGF-1 [1.6 (95% CI: 1.3, 2.1), *p* < 0.001 and 2.1 (95% CI: 1.7, 2.8), *p* < 0.001]; IGFBP3 [1.3 (95% CI: 1.1, 1.5), *p* = 0.002 and 1.7 (95% CI: 1.5, 2.0), *p* < 0.001], respectively. There were significant decreases in the molar excess of sOBR/total leptin in both the MAM and SAM groups [0.5 (95% CI: 0.4, 0.8), *p* < 0.001] and 0.5 (95% CI: 0.3, 0.7), *p* < 0.001], respectively (Table 4).

**Table:** Table 4. Hormone changes over time by nutritional group

Hormones	MAM Geometric mean ratio (95% CI)			SAM Geometric mean ratio (95% CI)			*p*^b^
	Days 14 vs. 0	Days 28 vs. 0	*p*^a^	Days 14 vs. 0	Days 28 vs. 0	*p*^a^	
Leptin, ng/ml	1.6 (1.1, 2.4)	1.1 (0.8, 1.6)	0.002	1.8 (1.3, 2.6)	1.6 (1.1, 2.3)	<0.001	0.18
sOBR, ng/ml	0.88 (0.8, 0.9)	1.0 (0.9, 1.1)	0.02	0.8 (0.7, 0.9)	0.8 (0.7, 0.9)	<0.001	0.02
Molar excess sOBR/leptin	0.5 (0.4, 0.8)	0.8 (0.6, 1.3)	<0.001	0.5 (0.3, 0.7)	0.5 (0.3, 0.7)	<0.001	0.04
Total ghrelin, ng/ml	0.9 (0.7, 1.1)	0.8 (0.6, 0.9)	0.007	0.7 (0.6, 0.9)	0.7 (0.6, 0.9)	<0.001	0.11
IGF-1, ng/ml	1.6 (1.3, 2.1)	1.3 (1.0, 1.7)	<0.001	2.1 (1.7, 2.8)	2.2 (1.7, 2.8)	<0.001	0.001
IGFBP3, ng/ml	1.3 (1.1, 1.5)	1.1 (1.0, 1.3)	0.002	1.7 (1.5, 2.0)	1.5 (1.3, 1.7)	<0.001	<0.001
Molar ratio IGF1:IGFBP3	1.3 (1.1, 1.6)	1.1 (1.0, 1.5)	0.005	1.2 (1.0, 1.5)	1.5 (1.2, 1.8)	<0.001	0.02
C-peptide, pM/l	1.1 (0.7, 1.8)	1.2 (0.7, 2.0)	0.69	0.9 (0.5, 1.5)	0.9 (0.6, 1.6)	0.91	0.70
Insulin, µIU/ml	0.8 (0.3, 2.1)	1.4 (0.5, 3.5)	0.40	1.9 (0.8, 4.7)	1.6 (0.7, 4.0)	0.16	0.23
Cortisol, µ/dl	0.9 (0.6, 1.3)	0.9 (0.6, 1.3)	0.64	0.7 (0.5, 1.0)	0.8 (0.6, 1.1)	0.03	0.47

a Bonferroni’s-adjusted test assessing differences over time.

b Wald test assessing interaction between nutritional group and time.

### Hormonal correlates of weight gain

The significant correlations between many of the hormonal measurements introduced confounding into the multivariable analysis; therefore, univariable analyses are reported. Changes in WAZ among the SAM and MAM groups combined were positively correlated with insulin (*F*-ratio 7.8, *p* = 0.006) and C-peptide (*F*-ratio 12.2, *p* < 0.001) but not with any of the other hormones or their binding proteins. Surprisingly, the association with leptin did not reach statistical significance (*p* = 0.07).

### Hormonal predictors of weight gain

The change in WAZ over the 28 days of active intervention (combined MAM and SAM) was predicted by Day 0 C-peptide (*F*-ratio 5.4, *p* = 0.03) and cortisol (*F*-ratio 5.0, *p* = 0.03) (both were positive associations) but not by any of the other hormonal indices or by salivary CRP. In multivariable analysis with both C-peptide and cortisol, the predictive value of C-peptide strengthens (*F*-ratio 7.6, *p* = 0.009) and it predicted 13.9% of the variance in weight recovery (for further details, see Supplementary Material).

## DISCUSSION

With optimal nutrition interventions, we expected a rapid and sustained rise in growth-promoting hormones and decline in pro-inflammatory hormones [8, 11] However, our study shows that in this setting, the significant part of nutritional recovery occurs in the first 2 weeks of nutritional rehabilitation but is variable between children. Insulin and C-peptide were the only hormones that were correlated to changes in WAZ in malnourished children and to a lesser extent cortisol.

Even in the modest samples of MAM and SAM children studied here, we noted a range spanning >2 *z*-scores in weight (WAZ) in response to the interventions; some children even deteriorated over the 28 days. With an increasing move towards treating uncomplicated cases of SAM in the community [3], it would be useful to identify predictors of response to therapy to guide the triaging of patients between inpatient and outpatient care protocols. Of all the anthropometric, health and biochemical indices tested, only C-peptide and baseline cortisol predicted WAZ gain over 28 days. We interpret the cortisol result as indicating that these children were more acutely sick at baseline and once brought into clinical care made the fastest response. Children had characteristic C-peptide values suggesting the possibility that differences in insulin production represent a constitutive determinant of the propensity to store energy and nutrients when available. Nonetheless, C-peptide only predicted 13.9% of the variance in recovery rates and attempts to combine this with other measures yielded no significant improvement in the prediction.

Previous studies have also shown that at the end of nutritional rehabilitation, leptin increases in the undernourished children, despite modest weight gain, sometimes reaching 166% of levels observed in well-nourished children [8, 9, 11, 13]. Somewhat surprisingly, we did not find such marked changes in our study possibly because almost all the children were on oral feeds from the onset of their nutritional rehabilitation or supplementation, hence resulting in a more natural course of nutritional recovery. We also found that sOBR and the molar excess of sOBR decreased during nutritional rehabilitation as did Stein *et al.* [11]; however, the levels of sOBR and the molar excess of sOBR did not drop below the levels observed in controls, as they found, again a possible reflection on the difference in energy-regulating hormone responses with the different modes of feeding during nutritional rehabilitation (nasogastric vs. oral) [3, 11].

Programmatically, MUAC is increasingly used in monitoring nutritional recovery [17]. At the time of the study WAZ that was more routinely monitored in the clinical settings in the Gambia and as MUAC correlated well with it, we used WAZ in our analyses of nutritional recovery.

This study had a number of limitations. Our sample size was modest in recognition of our ethical responsibilities in studying young children but had been validated as informative by a prior pilot study. Nonetheless, our findings confirm and extend our understanding of the endocrine changes observed in other populations recovering from malnutrition. We also excluded malnourished children who were severely unwell and those who were HIV infected; therefore, our findings can only be generalized to malnourished children with few or no complications. This is both a limitation and a strength, as the primary intention of this study was to identify possible prognostic indicators that would guide treatment decisions and children with complications necessitate inpatient care, so the treatment pathway is already established. None of the children in the study had kwashiorkor, and we were therefore unable to make comparisons of the hormonal changes between children with marasmus and kwashiorkor.

## CONCLUSION

In rural Gambian children, growth and energy-regulating hormones do not explain the high variability of responses to nutritional rehabilitation. Insulin and C-peptide were the variables most strongly associated with WAZ gain, and C-peptide was the only variable for which baseline values predicted the response to nutritional rehabilitation but would not be a useful prognostic tool in isolation.

## Supplementary Material

Supplementary Figure S1

Supplementary Data
