# Supplementary figures and images for: Hormonal Correlates and Predictors of Nutritional Recovery in Malnourished African Children
Source: J Trop Pediatr. 2017 Oct 30;64(5):364–72. doi: 10.1093/tropej/fmx075 (PMC6166213; doi:10.1093/tropej/fmx075)

## Slide 1
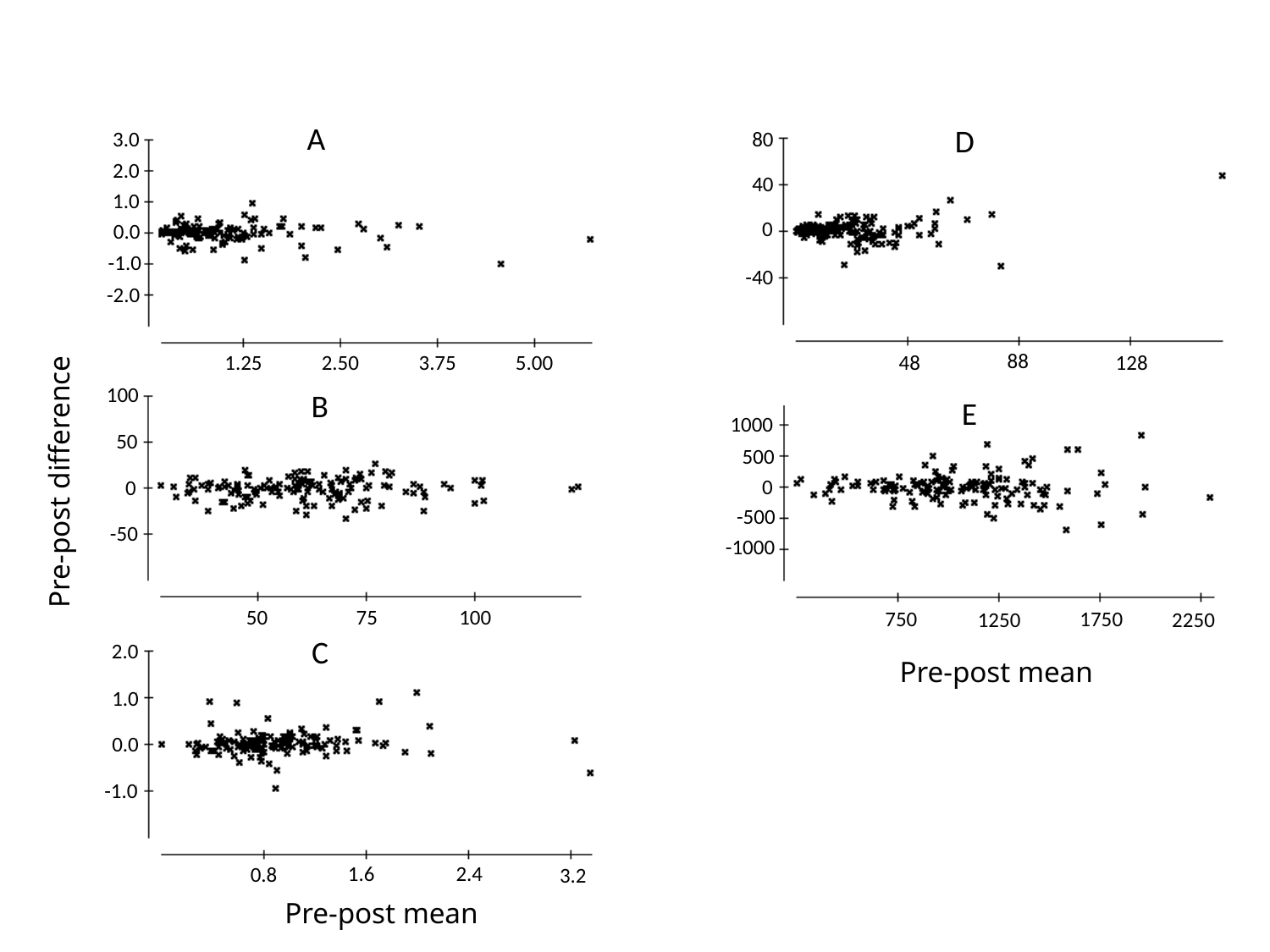

A
D
3.0
80
2.0
40
1.0
0
0.0
-1.0
-40
-2.0
88
3.75
5.00
48
128
1.25
2.50
100
B
E
1000
50
500
Pre-post difference
0
0
-500
-50
-1000
100
75
50
750
1750
1250
2250
C
2.0
Pre-post mean
1.0
0.0
-1.0
1.6
2.4
0.8
3.2
Pre-post mean

Supplement: Supplementary Figure S1 [file fmx075_supp_fig_1.pptx]
