# Supplementary material for: Hormonal Correlates and Predictors of Nutritional Recovery in Malnourished African Children
Source: J Trop Pediatr. 2017 Oct 30;64(5):364–72. doi: 10.1093/tropej/fmx075 (PMC6166213; doi:10.1093/tropej/fmx075)
Supplement: Supplementary Data [file fmx075_supplementary_material.docx]

**Hormonal Correlates and Predictors of Nutritional Recovery in Malnourished African Children**

Helen M Nabwera ^1,2^, Robin M Bernstein^3^, Schadrac Agbla ^1,2^, Sophie E Moore^1, 4^, Momodou K Darboe^1^, Mariama Colley^1^, Amadou T Jallow^1^, Richard Bradbury^1^, Jennifer Karafin^3^, Tony J Fulford ^1,2^ and Andrew M Prentice ^1,2^

1. Medical Research Council Unit The Gambia, P. O. Box 273, Banjul, The Gambia
2. Department of Population Health, London School of Hygiene and Tropical Medicine, Keppel street, London, WC1E 7HT, United Kingdom
3. Department of Anthropology, University of Colorado at Boulder, 1350 Pleasant Street
   Hale Science 350, 233 UCB Boulder, CO 80309-0233, USA
4. Division of Women’s Health, King’s College London, 10^th^ floor North Wing, St Thomas’ Hospital, Westminster Bridge Road, London, SE1 7EH, United Kingdom

**Supplementary material- contents**

1. Supplementary background
2. Supplementary methods
3. Supplementary results
4. Supplementary figure
5. Supplementary references
6. Supplementary legends
7. **Supplementary background**

Insulin-like-growth factor-1 and 2 (IGF-1 and 2) are the primary growth-promoting factors from fetal life until 6 months of age [1]. Thereafter the growth hormone (GH)-IGF-1 axis, and its interplay with the key insulin-like-growth factor binding protein-3 (IGFBP3), play an important role in regulating and coordinating postnatal growth [2]. Leptin is a pleiotropic hormone that plays key roles in energy homeostasis, appetite, immune regulation, modulation of intestinal barrier function and haematopoiesis [3]. Soluble leptin receptor (sOB-R) is the primary leptin-binding protein in human blood [4]. In the first years of life, sOB-R is detectable in remarkably high concentrations and levels decline continuously thereafter [5]. Ghrelin is an orexigenic hormone that can also stimulate the release of GH, and modulates digestive properties, sleep and muscle integrity [6]. Paradoxically, ghrelin levels are significantly higher in severely wasted children compared to controls, even in the context of poor appetite but decline with nutritional rehabilitation [7, 8].

1. **Supplementary methods**

*Study population*

At presentation, children were assessed and treated for any medical conditions by a paediatrician or medical officer. A medical and dietary history (including breastfeeding practices), sociodemographic profile, and physical examination was completed at enrolment.

SAM was defined by one of three criteria: 1) WHZ less than -3; and/or 2) mid upper arm circumference (MUAC) less than 115 mm; or 3) bilateral pedal oedema with other clinical features of kwashiorkor [9]. MAM was defined as WHZ less than between -2 and -3 [10].

*Nutritional composition of WHO-F75*

WHO F-75 is used in Phase 1 of the treatment of children over 6 months of age, suffering from severe acute malnutrition (SAM). It contains 75 kcalories and 0.9 grams of protein per 100 ml. Ingredients include concentrated milk powder, food oil and dextrin vitamin complexes to provide a reduced amount of proteins, fats and sodium, but rich in carbohydrates. [11]

*Study interventions*

The SAM children were initially managed in the nutritional rehabilitation unit (NRU) at MRC Keneba. Their care was supervised by the study nurse and 3 auxiliary nurses supported by a medical officer and paediatrician. On admission, they received F-75 (starter formula) then, F100 (catch up formula) and subsequently, Plumpy’Nut (Nutriset) ready-to-use-therapeutic food (RUTF) once their appetite and clinical condition had improved. During the initial stabilisation phase, all children received a 1 week course of oral or intravenous antibiotics. The children were fed orally from the outset, which most of them tolerated. Only four children who did not initially tolerate the oral feeds had nasogastric feeds for 24-48 hours. All the children over 1 year of age received oral mebendazole 250 mg on admission for treatment for parasitic infections. Children who presented with diarrhoea received oral zinc 20 mg per day for 10-14 days and the anaemic children with SAM also received one iron/folic acid combined tablet (Iron 60mg, Folate 400 µg), if <10kg and two tablets if >10kg, every two weeks after completing treatment for all the acute infections according to the Gambian guidelines. Children were discharged home from the NRU to the community management of malnutrition programmes (CMAM) when they were able to tolerate oral feeds including Plumpy’Nut and were gaining weight. Carers of children with MAM received nutritional counselling from the study nurse and received 28 days’ worth of RUTF. Carers of the controls received nutritional counselling from the study nurse but the children did not receive any nutritional supplements. All the children in the study had anthropometric measurements done by the study field workers on alternate days from Day 0-28, then at 6 months from baseline.

*Biological sample collection and analysis*

A maximum of 5ml of blood per child was collected at each time point. Blood samples were collected at 10am (pre-meal) and 1 hour later (post-meal), to account for potential circadian variation in all hormones measured. The saliva samples were collected using SalivaBio’s Children’s Swab (Salimetrics, Pennsylvania) and stored at -70°C. Blood samples were placed immediately on ice and processed promptly, and plasma stored at -70° until analysis. The saliva samples were collected using SalivaBio’s Children’s Swab (Salimetrics, Pennsylvania) and stored at -70°C. Samples were shipped on dry ice to the University of Colorado at Boulder, USA for analysis.

The assays that used for the analysis of leptin, leptin soluble receptor (sOBR), IGF-1 and IGFBP3 were from R&D Systems (R&D Systems, Minneapolis, USA) [12-15], for total ghrelin Merck Millipore (Merck Millipore, Darmstadt, Germany) [14], for cortisol and C-peptide ALCPO (ALPCO, New Hampshire, USA) [17, 18] and for salivary C-reactive protein (CRP) from Salimetrics (Salimetrics, Pennsylvania, USA) [19]. These assays use the quantitative sandwich enzyme-linked immunosorbent assay technique (ELISA). Monoclonal antibodies specific for the antigens had been pre-coated onto their respective microplates. Standards and samples were pipetted into the wells and any respective antigens present were bound by the immobilized antibody. After washing away any unbound substances, enzyme-linked monoclonal antibodies (horseradish peroxidise enzyme in the total ghrelin and salivary assays) specific for the respective antigens were added to the wells. Following a wash to remove any unbound antibody-enzyme reagent, a substrate solution was added to the wells and colour developed in proportion to the amount of antigen bound in the initial step. The colour development was stopped and the intensity of the colour was measured spectrophotometrically and compared to reference standards [12-19]. The intra and inter-assay coefficients of variation (CV) were 3% and 4.2% for leptin, 2.2% and 5.3% for sOBR, 4.3% and 8.3% for IGF-1, 5% and 6.5% for IGBP3, 3.9% and 4.6% for total ghrelin and 3.3% and 7.6% for C-peptide, 6.3% and 10.4% for cortisol, 9.6%, 10.6 % for insulin and 4.7% for salivary CRP respectively.

Clean catch urine samples (obtained when a child passes urine directly into a sterile pot after their perineum has been cleaned, in our case with medicated soap and clean water) [20], were collected and stored in a laboratory refrigerator at temperatures between 2-8°C. Analysis of urine was done using Combur 9 test strips (Roche Diagnostics Limited, Switzerland) urine and the results were interpreted using a coloured scale [21]. A urine dipstick result that was positive for nitrites and leucocytes; nitrites and blood or leucocytes and blood was reported as a urine infection. The urine dipstick analyses were performed within 12 hours of collecting sterile urine samples.

*Anthropometry*

Lengths were measured on a Raven Kiddimetre (Raven Equipment, Great Dunmow, Essex, UK). Weight measurements were done with infants unclothed and recorded to the nearest 10 grams using electronic Seca 336 high precision portable baby weighing scales. All the weighing scales were calibrated weekly. The MUAC was recorded to the nearest millimetre using MUAC tapes. The knee heel length was measured using a knenometer (Chasmors Ltd, London, UK) and recorded to the nearest millimetre.

*Statistical analysis*

A mixed effects model was used to assess for differences in hormone levels between nutritional groups at baseline and over time, allowing for interaction between nutritional groups and time points, adjusted for age and gender, which accounted for repeated measurements in each individual child i.e. pre- and post-prandial levels and over time. A piecewise linear random slope model was used to assess the change in weight, MUAC, knee heel, WHZ, weight-for-age Z-scores (WAZ) and height-for-age Z-scores (HAZ) over time and allow for variation in growth rate between individual children. We considered three time intervals: 0-14 days, 14-28 days and 29-180 days. A random slope was allowed for each time interval. The Wald test was used to test for interaction between time and nutritional group at both time intervals. Interaction terms, age at recruitment and sex were not included in the final model if there was no evidence at 5% level of significance.

1. **Supplementary results**

The median salivary CRP levels were lower in the controls (median [IQR] 2.9 [2.4, 4.1]) compared to MAM (median [IQR] 4.9 [2.8, 10.3]) and SAM (median [IQR] 5.6 [4.1, 9.9]); p=0.04. All the participants completed up to the 6-month follow-up visit. Children with SAM all received at least a week’s course of antibiotics.

The average length of admission for children with SAM was 11 days and 18 (90%) were discharged to the CMAM programmes on or before Day 14. Only 7 (35%) of children with SAM had achieved a WHZ >-2 on Day 28, but the majority 18 (90%) were no longer severely wasted i.e. WHZ >-3. Eleven (61%) of children with MAM had a WHZ >-2 on Day 28.

*Hormone status at baseline*

There was a very strong correlation (r <0.9 in all cases) between the pre- and post-prandial values with no significant deviation from the Y=X line (see Bland-Altman plots in Supplementary Figure 2). The strength of these correlations validates the precision of the assays and shows high discrimination ratios indicating that each of the indices has the potential to be good predictors of response.

The IGF-1: IGFBP3 molar ratio was only significantly lower in SAM compared to the controls (geometric mean ratio 0.7 [95% CI: 0.6, 0.9], p<0.05).

*Anthropometric changes over time*

At the lower end two SAM and eight MAM children showed a slight deterioration in WAZ despite the intensive intervention, whilst at the upper end three children gained close to +2 Z-scores (probably indicative of some recovery-associated water retention). There was a non-significant tendency for girls to recover better than boys (+0.70 vs +0.38 WAZ, p=0.07).

*Hormone changes over time*

For the SAM group, there were also significant decreases in sOBR (geometric mean ratio 0.8 [95%CI: 0.7, 0.9], p<0.001) and ghrelin (geometric mean ratio 0.7 [95%CI: 0.6, 0.9] p<0.001) over this time interval. However, from Day 0-28 of nutritional rehabilitation, the increases in total leptin, IGF-1 and IGFBP3 were only significant in the SAM group: total leptin (geometric mean ratio 1.6 [95%CI: 1.1, 2.3], p<0.001); IGF-1 (geometric mean ratio 2.2 [95%CI: 1.7, 2.8], p<0.001); and IGFBP3 (geometric means ratio 1.5 [95%CI: 1.3, 1.7], p<0.001). Conversely, there were significant decreases in total ghrelin in both MAM and SAM (geometric mean ratio 0.8 [95% CI: 0.6, 0.9], p=0.007 and 0.7 [95% CI 0.6, 0.9], p<0.001). In the SAM group, significant decreases were also found for sOBR (geometric mean ratio 0.8 [95%CI: 0.7, 0.9], p<0.001) and molar excess of sOBR: total leptin (geometric mean ratio 0.5 [95%CI: 0.3, 0.7], p<0.001).

There was weak evidence of declining cortisol levels from Day 0-28 in the SAM group (geometric mean ratio 0.8, [95%CI: 0.6, 1.1], p=0.03).

1. **Supplementary discussion**

In line with our findings, prior observational studies in low income countries have found that at baseline, SAM children have lower leptin, insulin, IGF-I and IGFBP-3; and higher basal cortisol, GH, soluble leptin binding receptor (sOBR) and IGFBP-1 compared to their non-malnourished counterparts [8, 22-24]. It has been hypothesised that low levels of leptin may stimulate the hypothalamic-pituitary-adrenal and hypothalamic–pituitary-growth hormone axes to maintain high cortisol and GH levels for lipolysis to provide fuel for the brain and other vital organs during nutritional deprivation, whilst maintaining low levels of IGF-1 and insulin until the onset of nutritional recovery [23-26]. These normalize to the levels in non-malnourished children within 2 weeks of intensive nutritional rehabilitation associated with rapid weight gain [8, 22-24].

A recent pilot study of children aged 18±4m being rehabilitated from SAM in our centre in rural Gambia, with age and sex matched community controls, found that in both groups a significant postprandial rise in leptin levels was found (Nweneka, Prentice *et al*, unpublished). We hypothesised, on the basis of Stein’s prior finding of very high sOBR: leptin ratios in malnutrition [24], that this rapid rise of postprandial leptin was due to a circulating reservoir of leptin bound to the soluble binding receptor (sOBR) that is released into the circulation acutely with feeding. As no other studies, have shown acute post-prandial rises in plasma leptin we were concerned that the initial study resulted from a methodological artefact and sought replication in the current study. Our concerns were validated as we failed to replicate an acute leptin response. Although there was no evidence of an immediate effect of feeding on the hormone levels during nutritional rehabilitation, our other findings on the more chronic responses of leptin and sOBR were consistent.

The measurement of the knee heel length on alternate days in children aged 6-24 months old, was primarily done to assess short term linear growth with greater precision than the standard length/height measurements. This is because anecdotally in our setting, repeated length/height measurements are often not reliable in children in this age group. Indeed, we found that over time (6 months), the knee heel length measurements were more reliable than the length/height measurements. However, in neonates, the knee heel length was found to be less reproducible than crown heel length as an assessment of linear growth [27]. However, in older children (3-16 years), the correlation between knee heel length was found to be a good predictor of short term linear growth but not annual linear growth, hypothesised as attributable to the variable growth rates of different types of the skeleton [28].

Note that growth in this population is generally poor so that, while the control group can be considered better nourished than SAM or MAM, they do not represent normal healthy growth according to international reference growth curves. WHZ scores against an internal reference generated using pooled anthropometric data from the Keneba database were: Controls 0.0, MAM -1.0 and SAM -1.7.

1. **Supplementary figure (separate file attached)**
2. **Supplementary references**
3. Karlberg J, Albertsson-Wikland K, Kwan CW, Chan FY: Early spontaneous catch-up growth. Journal of pediatric endocrinology & metabolism: JPEM 2002, 15 Suppl 5:1243-1255.
4. Mavalli MD, DiGirolamo DJ, Fan Y, Riddle RC, Campbell KS, van Groen T, Frank SJ, Sperling MA, Esser KA, Bamman MM et al: Distinct growth hormone receptor signaling modes regulate skeletal muscle development and insulin sensitivity in mice. The Journal of clinical investigation 2010, 120(11):4007-4020.
5. Faggioni R, Feingold KR, Grunfeld C: Leptin regulation of the immune response and the immunodeficiency of malnutrition. The FASEB journal: official publication of the Federation of American Societies for Experimental Biology 2001, 15(14):2565-2571.
6. Lammert A, Kiess W, Bottner A, Glasow A, Kratzsch J: Soluble leptin receptor represents the main leptin binding activity in human blood. Biochem Biophys Res Commun 2001, 283 (4): 982-8.
7. Ong KK, Ahmed ML, Sherriff A, Woods KA, Watts A, Golding J, Dunger DB Cord blood leptin is associated with size at birth and predicts infancy weight gain in humans. ALSPAC Study Team. Avon Longitudinal Study of Pregnancy and Childhood. J Clin Endocrinol Metab 84 (3): 1145-8.
8. Tanaka-Shintani M, Watanabe M: Distribution of ghrelin-immunoreactive cells in human gastric mucosa: comparison with that of parietal cells. Journal of gastroenterology 2005, 40(4):345-349.
9. Altinkaynak S, Selimoglu MA, Ertekin V, Kilicarslan B: Serum ghrelin levels in children with primary protein-energy malnutrition. Pediatrics international : official journal of the Japan Pediatric Society 2008, 50(4):429-431.
10. Bartz S, Mody A, Hornik C, Bain J, Muehlbauer M, Kiyimba T, Kiboneka E, Stevens R, Bartlett J, St Peter JV et al: Severe acute malnutrition in childhood: hormonal and metabolic status at presentation, response to treatment, and predictors of mortality. The Journal of clinical endocrinology and metabolism 2014, 99(6):2128-2137.
11. WHO: Pocket book of hospital care for children Guidelines for the management of common illnesses with limited resources, 2 edn; 2013.
12. Golden MH GY: Integrated Management of Acute Malnutrition. In.; 2012: 204.
13. Nutriset. Therapeutic milk F-75. 2016; <http://www.nutriset.fr/index.php?id=88>. Accessed 27/01/2017.
14. Quantikine ELISA Human Leptin Immunoassay

<https://resources.rndsystems.com/pdfs/datasheets/dlp00.pdf> Accessed 27/01/2017

1. Quantikine ELISA Human Leptin sR Immunoassay

<http://www.rndsystems.com/pdf/dobr00.pdf> Accessed 27/01/2017

1. Quantikine ELISA Human IGF-I Immunoassay

<https://resources.rndsystems.com/pdfs/datasheets/dg100.pdf> Accessed 27/01/2017

1. Quantikine ELISA Human IGFBP-3 Immunoassay

<https://resources.rndsystems.com/pdfs/datasheets/dgb300.pdf> Accessed 27/01/2017

1. Human ghrelin (total) ELISA kit 96-Well Plate (Cat. # EZGRT-89K)

<https://www.merckmillipore.com/INTL/en/product/Human-Ghrelin-%28total%29-ELISA,MM_NF-EZGRT-89K?ReferrerURL=https%3A%2F%2Fwww.google.com%2F&bd=1>

Accessed 27/01/2017

1. ALPCO Cortisol ELISA

<https://www.alpco.com/pdfs/11/11-CORHU-E01.pdf> Accessed 27/01/2017

1. ALPCO C-peptide ELISA

<https://www.alpco.com/pdfs/80/80-CPTHU-E01.1.pdf> Accessed 27/01/2017

1. Salimetrics Salivary C-Reactive Protein ELISA kit

<https://www.salimetrics.com/assets/documents/1-3302.pdf> Accessed 27/01/2017

1. Tosif S, Baker A, Oakley E, Donath S, Babl FE. Contamination rates of different urine collection methods for the diagnosis of urinary tract infections in young children: an observational cohort study. J Paediatr Child Health 2012;48(8):659-64.
2. Combur 9 test strips, Roche Diagnostics

<http://www.roche-diagnostics.ch/content/dam/corporate/roche-dia_ch/documents/broschueren/professional_diagnostics/urindiagnostik/12254620001_EN_EA_Compendium-of-urinanalysis_Brosch%C3%BCre_EN.pdf> Accessed 27/01/2017

1. Doherty CP, Crofton PM, Sarkar MA, Shakur MS, Wade JC, Kelnar CJ, Elmlinger MW, Ranke MB, Cutting WA: Malnutrition, zinc supplementation and catch-up growth: changes in insulin-like growth factor I, its binding proteins, bone formation and collagen turnover. Clinical endocrinology 2002, 57(3):391-399.
2. Palacio AC, Perez-Bravo F, Santos JL, Schlesinger L, Monckeberg F: Leptin levels and IgF-binding proteins in malnourished children: effect of weight gain. Nutrition 2002, 18(1):17-19.
3. Stein K, Vasquez-Garibay E, Kratzsch J, Romero-Velarde E, Jahreis G: Influence of nutritional recovery on the leptin axis in severely malnourished children. The Journal of Clinical Endocrinology and Metabolism 2006, 91(3):1021-1026.
4. Soliman AT, ElZalabany MM, Salama M, Ansari BM: Serum leptin concentrations during severe protein-energy malnutrition: correlation with growth parameters and endocrine function. Metabolism clinical and experimental 2000, 49(7):819-825.
5. Kilic M, Taskin E, Ustundag B, Aygun AD: The evaluation of serum leptin level and other hormonal parameters in children with severe malnutrition. Clinical biochemistry 2004, 37(5):382-387.
6. Griffin IJ, Pang NM, Perring J, Cooke RJ: Knee-heel length measurement in healthy preterm infants. Archive of Diseases in Childhood, Fetal and Neonatal Edition 1999, 81: F50-55.
7. **Supplementary figure Legends**
   1. Pre- and post-prandial differences in leptin (ng/ml)
   2. Pre- and post-prandial differences in soluble leptin receptor (sOBR) (ng/ml)
   3. Pre- and post-prandial differences in total ghrelin (ng/ml)
   4. Pre- and post-prandial differences in insulin like growth factor 1 (IGF-1) (ng/ml)
   5. Pre- and post-prandial differences in insulin like growth factor binding protein 3 (IGFBP3) (ng/ml)
